# Prognostic value of CAD-RADS classification by coronary CTA in patients with suspected CAD

**DOI:** 10.1186/s12872-021-02286-x

**Published:** 2021-10-03

**Authors:** Zengfa Huang, Shutong Zhang, Nan Jin, Yun Hu, Jianwei Xiao, Zuoqin Li, Yang Yang, Ruihong Sun, Zheng Wang, Xiang Li, Yuanliang Xie, Xiang Wang

**Affiliations:** 1 Department of Radiology, The Central Hospital of Wuhan, Tongji Medical College, Huazhong University of Science and Technology, 26 Shengli Avenue, Jiangan, Wuhan, 430014 Hubei China; 2 Department of Geriatrics, Union Hospital, Tongji Medical College, Huazhong University of Science and Technology, Wuhan, 430022 China

**Keywords:** Coronary artery disease, Coronary computed tomography angiography, CAD-RADS, Coronary stenosis, Prognosis

## Abstract

**Background:**

The study sought to compare Coronary Artery Disease Reporting and Data System (CAD-RADS) classification with traditional coronary artery disease (CAD) classifications and Duke Prognostic CAD Index for predicting the risk of all-cause mortality in patients with suspected CAD.

**Methods:**

9625 consecutive suspected CAD patients were assessed by coronary CTA for CAD-RADS classification, traditional CAD classifications and Duke Prognostic CAD Index. Kaplan–Meier and multivariable Cox models were used to estimate all-cause mortality. Discriminatory ability of classifications was assessed using time dependent receiver-operating characteristic (ROC) curves and The Hosmer–Lemeshow goodness-of-fit test was employed to evaluate calibration.

**Results:**

A total of 540 patients died from all causes with a median follow-up of 4.3 ± 2.1 years. Kaplan–Meier survival curves showed the cumulative events increased significantly associated with CAD-RADS, three traditional CAD classifications and Duke Prognostic CAD Index. In multivariate Cox regressions, the risk for the all-cause death increased from HR 0.861 (95% CI 0.420–1.764) for CAD-RADS 1 to HR 2.761 (95% CI 1.961–3.887) for CAD-RADS 4B&5, using CAD-RADS 0 as the reference group. The relative HRs for all-cause death increased proportionally with the grades of the three traditional CAD classifications and Duke Prognostic CAD Index. The area under the time dependent ROC curve for prediction of all-cause death was 0.7917, 0.7805, 0.7991for CAD-RADS in 1 year, 3 year, 5 year, respectively, which was non-inferior to the traditional CAD classifications and Duke Prognostic CAD Index.

**Conclusions:**

The CAD-RADS classification provided important prognostic information for patients with suspected CAD with noninvasive evaluation, which was non-inferior than Duke Prognostic CAD Index and traditional stenosis-based grading schemes in prognostic value of all-cause mortality. Traditional and simplest CAD classification should be preferable, given the more number of groups and complexity of CAD-RADS and Duke prognostic index, without using more time consuming classification.

**Supplementary Information:**

The online version contains supplementary material available at 10.1186/s12872-021-02286-x.

## Background

Cardiovascular disease (CVD) remains the leading cause of premature mortality and rising health care costs [1, 2]. A multinational collaborative research study recently reported the prevalent case of CVD nearly doubled and the number of CVD deaths increased 6.5 million in the world from 1990 to 2019 [3]. Coronary computed tomography angiography (CTA) has been proved to be reliable for triage patients with stable or acute chest pain of ischemic origin [4]. UK National Institute for Health and Clinical Excellence (NICE) and European Society of Cardiology (ESC) recommend coronary CTA as a first-line strategy for evaluation of patients presenting with non-acute chest pain [5] or with chest pain and low to intermediate likelihood of having obstructive coronary artery disease (CAD) [6]. It seems likely that coronary CTA will play an increasingly dominant role for the evaluation of suspected CAD based on these developments.

The Coronary Artery Disease-Reporting and Data System (CAD-RADS) is a new standardized method for classification of CAD, which is then integrated into patient-specific clinical care [7]. The international CONFIRM registry recently reported that CAD-RADS was associated with 5-year outcome in 5039 patients [8]. Another recent study revealed that CAD-RADS added incremental prognostic value beyond atherosclerotic cardiovascular disease (ASCVD) risk score and coronary artery calcification scores (CACS) with a median follow-up of 2 years [9]. However, this prognostic value of CAD-RADS has been reported only in the US and other western countries with a relative short term follow-up. Whether there is incremental prognostic value of CAD-RADS beyond existing traditional stenosis classifications or Duke Prognostic CAD Index in the Chinese population with long term follow-up is unknown. Thus, the aim of this study was to evaluate the prognostic value of CAD-RADS classifications to predict the risk of all-cause mortality in patients with suspected CAD compared with traditional stenosis classifications and Duke Prognostic CAD Index.

## Methods

This retrospective study was approved by the Central Hospital of Wuhan, Tongji Medical College, Huazhong University of Science and Technology. We confirmed that all methods were performed in accordance with the related guidelines and the principles of the Declaration of Helsinki. Written informed consent was waived because of its retrospective observational nature by Institutional Review Board of the Clinical Research Institute at The Central Hospital of Wuhan.

### Study population

**Fig. 1:**
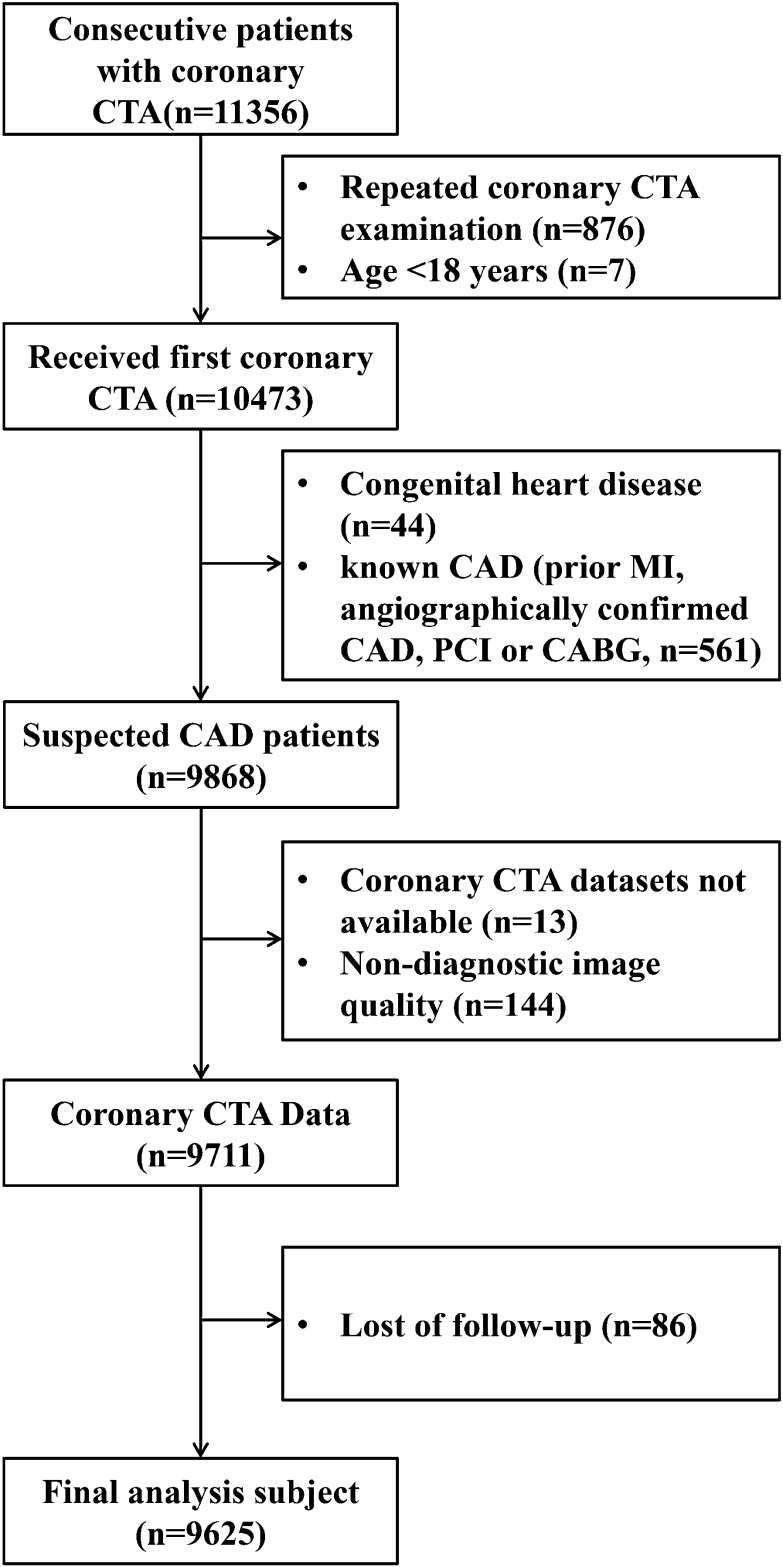
Flowchart illustrates exclusion criteria and final study population. *CTA* computed tomography angiography, *CAD* coronary artery disease, *MI* myocardial infarction

This study population consisted of 11,356 consecutive suspected CAD patients who underwent coronary CTA for evaluation from January 2012 to December 2019 (Fig. 1). Patients included in our analysis meet the following inclusion criteria: (1) adults who were 18 years old or older; (2) refer for coronary CTA using a ≥ 64-detector row scanner; (3) good quality images acquired that could be diagnosed with standardized reporting of segmental coronary stenosis [10]. The following exclusion criteria were used: (1) repeated coronary CTA examination in the database (n = 876) and patients younger than 18 years (n = 7); (2) patients had congenital heart disease (n = 44); (3) patients had known CAD (prior MI, angiographically confirmed CAD, PCI or CABG, n = 561); (4) Coronary CTA datasets were not available (n = 44) or non-diagnostic image quality (including insufficient image quality, motion of one or more vessels, bad contrast and wrong bolus timing, n = 144) and (5) loss of follow-up (n = 86). At last, a total of 9625 patients were included in the final analysis.

### Coronary CTA protocol, CAD-RADS, traditional CAD classifications and Duke Prognostic CAD Index definitions

Coronary CTA was performed using one of the four CT systems: Aquilion 64, Toshiba Medical Systems, Otawara, Japan; Philips Brilliance iCT, Philips Medical Systems, Best, the Netherlands; uCT 760, United Imaging, China; SOMATOM Definition AS, Siemens Healthineers, Germany which has been reported previously [11]. Heart rate control (HR ≥ 65 beats/min) was performed with beta-blockers before the scan. Scanning parameters were as following: tube voltage 120 kV, tube current 280–300 mAs. For contrast enhancement, 60–80 mL of iopromide (370 mgI/mL, Bayer Schering Pharma, Germany) followed by 30–40 mL of pure saline with a flow rate of 4–5 mL/s. The iodine contrast agent was automatically triggered into descending aorta of 100 HU threshold units. Then the scanning was performed during an inspiratory breath hold of 8–14 s after delay of 2 s. The reconstruction images were automatic send to one of the four workstations VITREA 2 (version 6.1, Vital Images, Inc, Minnesota, America), Intelligence space portal (Version 6.0.4, Philips Medical Systems, Best, the Netherlands), UIH Advanced Workstation (uWS-CT, R004, United Imaging Healthcare, Shanghai, China), SyngoVIA (Siemens Medical Solutions, Forchheim, Gemany).

First, according to the CAD-RADS consensus document [7], the standardized CAD-RADS classification was based on the highest degree of coronary stenosis and defined as follows: CAD-RADS 0: 0% stenosis, CAD-RADS 1: 1–24% stenosis, CAD-RADS 2: 25–49% stenosis, CAD-RADS 3: 50–69% stenosis, CAD-RADS 4A: 70–99% stenosis in 1 or 2 vessels, CAD-RADS 4B: Left main > 50% stenosis or 3-vessel disease, 70–99% stenosis, CAD-RADS 5: 100% stenosis or total occlusion. In order to restrict our analysis to patients without previously known CAD or revascularization, we did not include CAD-RADS modifiers to describe patients with stents (modifier S), or grafts (modifier G) in our analysis. Second, three traditional stenosis classifications and Duke Prognostic CAD Index were described as following [8, 9, 12]: Traditional CAD Classification 1, no CAD: 0% stenosis, mild CAD: 1–49% stenosis, moderate CAD: 50–69% stenosis in any major vessels/branch, severe CAD: ≥ 50% stenosis in LM or ≥ 70% stenosis in any major vessels/branch. Traditional CAD Classification 2, normal: 0% stenosis, mildly abnormal: 1–69% stenosis in any major vessels/branch, or 1–49% stenosis in LM, moderately abnormal: ≥ 70% stenosis in any major vessels/branch, severely abnormal: ≥ 70% stenosis in 2 or more vessel, or ≥ 50% stenosis in LM, or ≥ 70% stenosis in proximal left anterior descending (pLAD). Traditional CAD Classification 3, no CAD: 0% stenosis, nonobstructive CAD: 1–49% stenosis in any major vessels/branch, 1 vessel obstructive CAD: ≥ 50% stenosis in 1 major vessels/branch, 2 vessel obstructive CAD: ≥ 50% stenosis in 2 major vessels/branch, 3 vessel/LM obstructive CAD: ≥ 50% stenosis in 3 major vessels/branch, or ≥ 50% stenosis in LM. Duke Prognostic CAD Index, Duke CAD 0: 0% stenosis in all vessels, Duke CAD 1: 1–24% stenosis, or at most 1 with 25–49% stenosis, Duke CAD 2: ≥ 2 stenosis 25–49%, Duke CAD 3: 1 vessel with 50–69% stenosis, Duke CAD 4: 2 stenosis 50–69%, or 1 vessel with ≥ 70% stenosis, Duke CAD 5: 3 stenosis 50–69%, or 2 vessels with ≥ 70% stenosis, or pLAD stenosis ≥ 70%, Duke CAD 6: 3 vessels ≥ 70% stenosis, 2 vessels with ≥ 70% stenosis with pLAD, Duke CAD 7: LM stenosis ≥ 50%.

### Follow-up

The primary endpoint was all-cause mortality. Follow-up procedures were approved by our hospital’s institutional review board (the Institutional Review Board of the Clinical Research Institute at The Central Hospital of Wuhan). Death status was ascertained by querying the local Community Health Service Centers. For death outside of the city, event was determined through telephone call, or review of medical records. The deadline date of follow-up was December 31, 2020.

### Statistical analysis

Continuous variables were presented as mean ± SD. Categorical variables are presented as frequencies and percentages. We used Student’s *t* test for continuous variables between groups. Categorical variables were compared using a chi-square test or Fisher’s exact test as appropriate. Cox proportional hazards regression models were used to calculated time to death of all cause and hazard ratios (HR) with 95% confidence intervals (95% CI). Kaplan–Meier method was used to estimate cumulative event-free survival. Time dependent Receiver-operating characteristic (ROC) curves were used to evaluate the discriminatory value of traditional CAD classifications, Duke Prognostic CAD Index and CAD-RADS grading for the outcome. The calibration of models compares the agreement between the predicted and observed event rates. The Hosmer–Lemeshow goodness-of-fit test was employed to evaluate calibration. This test divided patients into deciles according to the risk scores of models and compared the predicted versus the observed rates of mortality. A significant *P* value indicates a lack-of-fit and suboptimal calibration. A two-tailed *P* < 0.05 was considered statistically significant. All statistical analyses were performed using Stata version 16 (StataCorp LP, College Station, Texas) and R statistical package (version 3.6.3, R foundation for Statistical Computing, Vienna, Austria).

## Results

### Baseline clinical and coronary CTA characteristics of the study

Overall, 9625 patients were included in the final analysis. Figure 1 showed the reason for exclusion. Of the 9625 patients, the average age was 59.8 ± 10.7 years, and 44.3% (4262 of 9619) were male, the baseline clinical characteristics and coronary CTA characteristics of the study population in each group based on three traditional CAD classifications, Duke Prognostic CAD Index or CAD-RADS classification were shown in Table 1.

**Table 1:** Baseline characteristics of the study population (N = 9625)

	All patients (N = 9625)	Survival patients (N = 9085)	Death (N = 540)	*P* value
Age (years)	59.8 (10.7)	59.2 (10.5)	69.4 (10.2)	< 0.001
Gender (male)	4262 (44.3)	3934 (43.3)	328 (60.7)	< 0.001
Traditional CAD classification 1				< 0.001
No CAD	4425 (46.0)	4310 (47.4)	115 (21.3)	
Mild CAD	3449 (35.8)	3236 (35.6)	213 (39.4)	
Moderate CAD	1008 (10.5)	920 (10.1)	88 (16.3)	
Severe CAD	743 (7.7)	619 (6.8)	124 (23.0)	
Traditional CAD classification 2				< 0.001
Normal	4425 (46.0)	4310 (47.4)	115 (21.3)	
Mildly abnormal	4457 (46.3)	4156 (45.7)	301 (55.7)	
Moderately abnormal	198 (2.1)	168 (1.8)	30 (5.6)	
Severely abnormal	545 (5.7)	451 (5.0)	94 (17.4)	
Traditional CAD classification 3				< 0.001
No CAD	4425 (46.0)	4310 (47.4)	115 (21.3)	
Nonobstructive CAD	3449 (35.8)	3236 (35.6)	213 (39.4)	
1 vessel obstructive CAD	991 (10.3)	916 (10.1)	75 (13.9)	
2 vessel obstructive CAD	371 (3.9)	310 (3.4)	61 (11.3)	
3 vessel/LM obstructive CAD	389(4.0)	313(3.4)	76(14.1)	
Duke CAD index				< 0.001
Duke CAD 0	4425 (46.0)	4310 (47.4)	115 (21.3)	
Duke CAD 1	2009 (20.9)	1918 (21.1)	91 (16.9)	
Duke CAD 2	1440 (15.0)	1318 (14.5)	122 (22.6)	
Duke CAD 3	750 (7.8)	698 (7.7)	52 (9.6)	
Duke CAD 4	339 (3.5)	292 (3.2)	47 (8.7)	
Duke CAD 5	347 (3.6)	296 (3.3)	51 (9.4)	
Duke CAD 6	133 (1.4)	112 (1.2)	21 (3.9)	
Duke CAD 7	182 (1.9)	141 (1.6)	41 (7.6)	
CAD-RADS				< 0.001
0	4425 (46)	4310 (47.4)	115 (21.3)	
1	325 (3.4)	317 (3.5)	8 (1.5)	
2	3124 (32.5)	2919 (32.1)	205 (38)	
3	1008 (10.5)	920 (10.1)	88 (16.3)	
4A	472 (4.9)	401 (4.4)	71 (13.1)	
4B&5	271 (2.8)	218 (2.4)	53 (9.8)	

*CAD* coronary artery disease, *CAD-RADS* Coronary Artery Disease-Reporting and Data System

### Estimating death from all causes

**Fig. 2:**
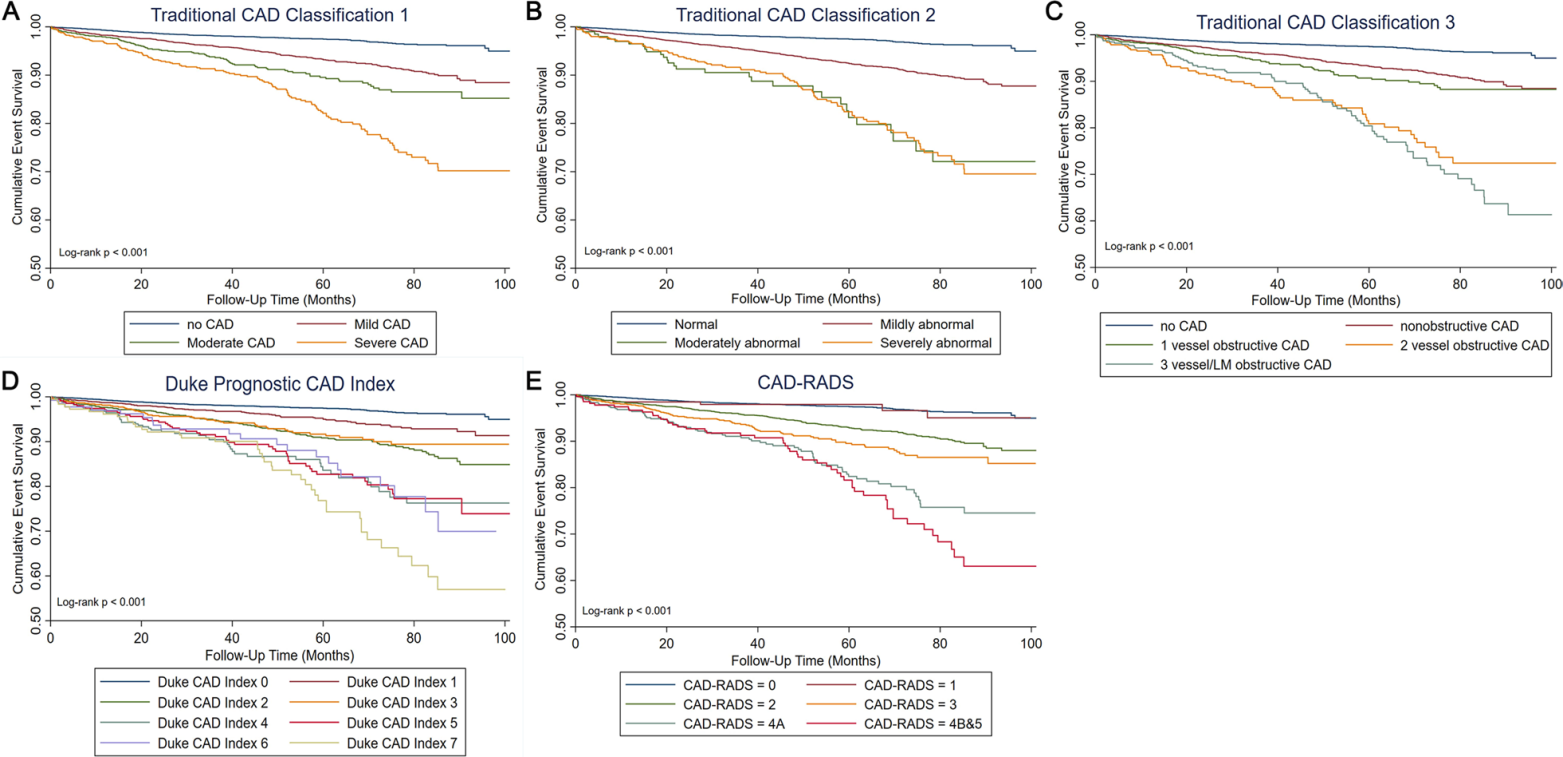
Cumulative event survival of follow-up by traditional CAD classifications, Duke prognostic CAD index and CAD-RADS score. *CAD* coronary artery disease, *CAD-RADS* Coronary Artery Disease-Reporting and Data System

A total of 540 patients died from all causes with a median follow-up of 4.3 ± 2.1 years. Kaplan–Meier survival curves showed the cumulative events increased significantly associated with CAD-RADS, three traditional CAD classifications and Duke Prognostic CAD Index (log-rank test, all *P *< 0.001, Fig. 2). The univariate Cox regression analysis showed that compared to CAD-RADS 0 group, CAD-RADS 1, 2, 3, 4A, 4B, 5 were significantly associated with all-cause mortality (all *P *< 0.001).

In multivariate Cox regressions that were adjusted for gender and age, the risk for the all-cause death increased from HR 0.861 (95% CI 0.420–1.764) for CAD-RADS 1 to HR 2.761 (95% CI 1.961–3.887) for CAD-RADS 4B&5, using CAD-RADS 0 as the reference group. The relative HRs for all-cause death increased proportionally with the grades of the three traditional CAD classifications and Duke Prognostic CAD Index (Table 2).

**Table 2:** Association of different classifications for CAD with all-causes mortality in the study

	Univariable cox		Multivariable cox	
	HR (95%)	*P* value	HR (95%)	*P* value
Traditional CAD classification 1				
No CAD	1 (ref)			
Mild CAD	2.508 (1.999–3.147)	< 0.001	1.376 (1.087–1.742)	0.008
Moderate CAD	3.829 (2.901–5.055)	< 0.001	1.572 (1.172–2.109)	0.003
Severe CAD	7.024 (5.449–9.053)	< 0.001	2.563 (1.950–3.370)	< 0.001
Traditional CAD classification 2				
Normal	1 (ref)			
Mildly abnormal	2.789 (2.250–3.458)	< 0.001	1.420 (1.132–1.782)	0.002
Moderately abnormal	7.045 (4.713–10.531)	< 0.001	2.825 (1.871–4.266)	< 0.001
Severely abnormal	7.015 (5.342–9.213)	< 0.001	2.465 (1.841–3.301)	< 0.001
Traditional CAD classification 3				
No CAD	1 (ref)			
Nonobstructive CAD	2.508 (1.999–3.147)	< 0.001	1.382 (1.092–1.750)	0.007
1 vessel obstructive CAD	3.243 (2.424–4.338)	< 0.001	1.406 (1.036–1.908)	0.029
2 vessel obstructive CAD	7.403 (5.426–10.100)	< 0.001	2.689 (1.937–3.734)	< 0.001
3 vessel/LM obstructive CAD	8.211 (6.145–10.971)	< 0.001	2.756 (2.024–3.752)	< 0.001
Duke CAD index				
0	1 (ref)			
1	1.876 (1.425–2.470)	< 0.001	1.210 (0.915–1.601)	0.181
2	3.349 (2.595–4.320)	< 0.001	1.568 (1.200–2.049)	0.001
3	3.019 (2.175–4.189)	< 0.001	1.334 (0.949–1.875)	0.097
4	6.336 (4.512–8.898)	< 0.001	2.408 (1.689–3.433)	< 0.001
5	6.120 (4.401–8.511)	< 0.001	2.355 (1.667–3.326)	< 0.001
6	6.267 (3.936–9.979)	< 0.001	2.313 (1.438–3.719)	< 0.001
7	9.505 (6.654–13.578)	< 0.001	3.095 (2.127–4.505)	< 0.001
CAD-RADS				
0	1 (ref)			
1	1.250 (0.610–2.561)	0.541	0.861 (0.420–1.764)	0.682
2	2.610 (2.077–3.280)	< 0.001	1.412 (1.113–1.792)	0.004
3	3.828 (2.900–5.053)	< 0.001	1.577 (1.176–2.116)	0.002
4A	6.412 (4.770–8.620)	< 0.001	2.449 (1.792–3.347)	< 0.001
4B&5	8.049 (5.813–11.145)	< 0.001	2.761 (1.961–3.887)	< 0.001

Multivariable cox models were adjusted for patient age and sex

*CAD* coronary artery disease, *CAD-RADS* Coronary Artery Disease-Reporting and Data System

### Discriminatory ability of classifications

**Fig. 3:**
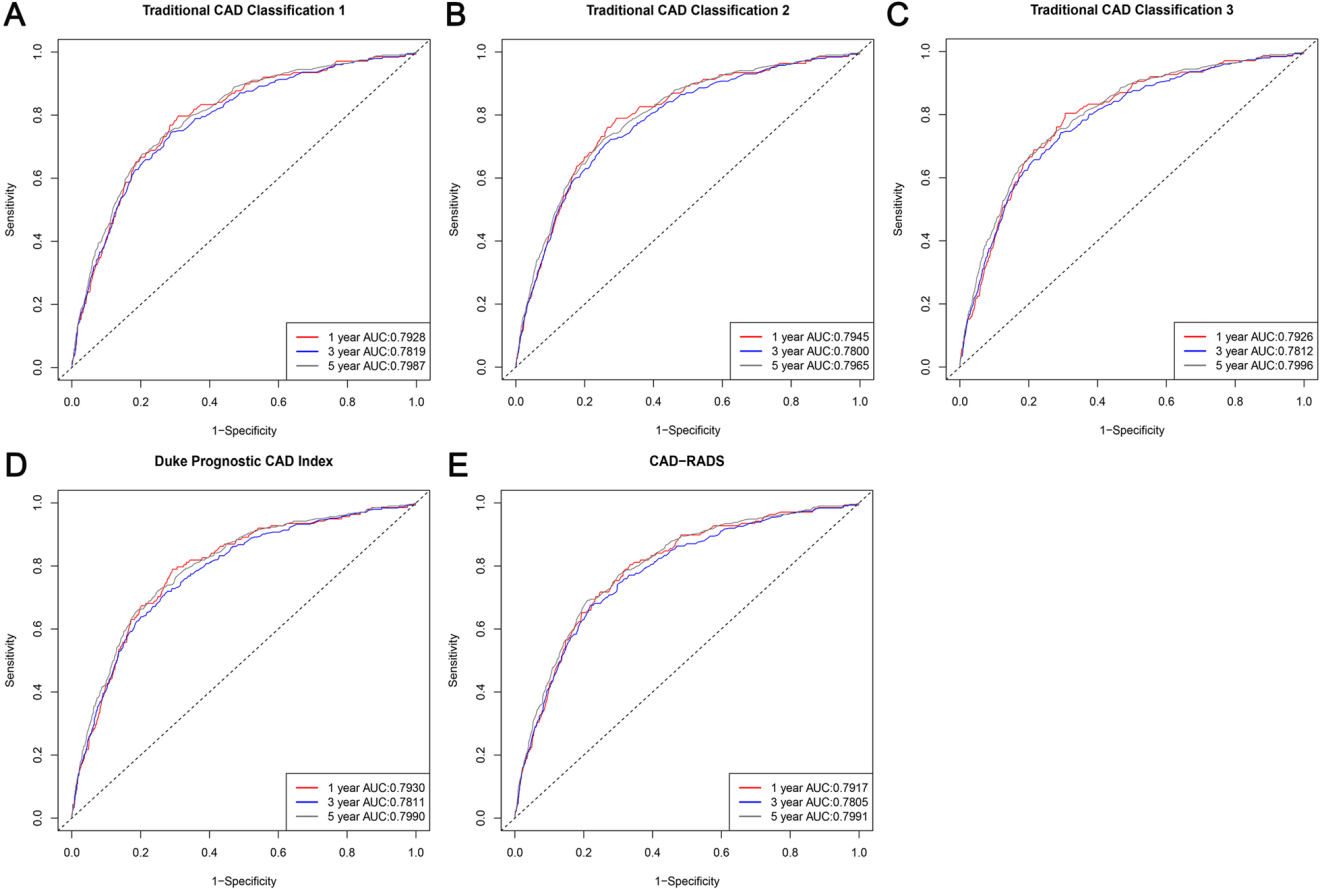
Time dependent ROC cures for prediction of all-cause mortality. *CAD* coronary artery disease, *CAD-RADS* Coronary Artery Disease-Reporting and Data System, *AUC* area under curve

The prognostic performance of CAD-RADS compared to the three traditional CAD classification of characterizing CAD extent/severity and Duke Prognostic CAD Index were shown in Fig. 3. The area under the time dependent ROC curve for prediction of all-cause death was 0.7917, 0.7805, 0.7991 for CAD-RADS in 1 year, 3 year, 5 year, respectively, which was similar compared to the traditional CAD classification 1 (0.7928 in 1 year, 0.7819 in 3 year, 0.7987 in 5 year; all *P* > 0.05), traditional CAD classification 2 (0.7945 in 1 year, 0.78 in 3 year, 0.7965 in 5 year; all *P* > 0.05), traditional CAD classification 3 (0.7926 in 1 year, 0.7812 in 3 year, 0.7996 in 5 year; all *P* > 0.05) and Duke Prognostic CAD Index (0.7930 in 1 year, 0.7811 in 3 year, 0.799 in 5 year; all *P* > 0.05). The five Classifications showed good calibration in the overall cohort (Hosmer–Lemeshow *P* value were 0.732, 0.71, 0.39, 0.505 and 0.91 for traditional CAD classification 1 traditional CAD classification 2, traditional CAD classification 3, Duke Prognostic CAD Index and CAD-RADS, respectively) (Additional file 1: Figure S1).

## Discussion

The present study examine a long-term prognosis associated with the novel CAD-RADS classification in the Chinese population, which were strongly predictive of all-cause death among suspected CAD patients in a real-world and were non-inferior to Duke Prognostic CAD Index and other coronary CTA prognostic classifications.

As a collaborative effort, CAD-RADS can now guide for clinical management with standardize CAD classifications after noninvasive imaging and may enhance communication between test abnormalities and optimal patient care. As a new classification system, high consistency of diagnosis is an essential prerequisite for its application. In a recent study [13], Inter-observer reproducibility of CAD-RADS categories was evaluation. The agreement among expert readers (ICC 0.925, 95% CI 0.884–0.954) and early career readers (ICC 0.904, 95% CI 0.852–0.941) were both excellent. However, the concordance for modifier “V” (high-risk plaque) is fair (*kappa* = 0.40), with poor-to-fair agreement for each of the high-risk plaque features. These findings were supported by another recent study [14]. The CAD-RADS category is the most important, regardless of the presence of a modifier [15]. Based on these results, the present study did not incorporated modifier (V) in the analysis.

From the large retrospective study, CAD-RADS grades were strongly associated with all-cause mortality, ranging from a 2- to fivefold increase in risk with progressively higher grades compared to a normal coronary CTA. We further found that CAD-RADS did not improve CAD risk discrimination for future events compared to the traditional CAD classifications and Duke Prognostic CAD Index. This findings was in keeping with the CONFIRM (Coronary CT Angiography Evaluation For Clinical Outcomes) registry study which has shown that increasing CAD-RADS scores were correlated with an increased risk of all-cause mortality up to a HR of 3.09 (95% CI 1.87–4.92) for patients with CAD-RADS 5 after adjusted for patient demographics and cardiac risk factors [8]. Analysis from the PROMISE (PROMISE [Prospective Multicenter Imaging Study for Evaluation of Chest Pain) study [9] showed that CAD-RADS had significantly higher discriminatory value than traditional CAD classifications (C-statistic 0.747 vs. 0.698–0.717; all *P* ≤ 0.001) which was different from the present study and the CONFIRM registry study findings. However, although with a randomized comparative effectiveness trial design from 193 sites, the relative smaller sample of 3840 patients were included in the analysis compared with the present study. Moreover, high-risk plaque (HRP) was added in the analysis and the agreement among expert readers for HRP (*kappa* = 0.56) is lower than that of ≥ 70% stenosis or left main ≥ 50% stenosis (*kappa* = 0.69). In addition, the definition of the endpoint was difference from the two studies that our primary outcome was all-cause mortality, yet nonfatal myocardial infarction was added in the CONFIRM registry study and myocardial infarction and hospitalization for unstable angina in the PROMISE study. The present study revealed that the patients with CAD-RADS 4B&5 classifications were at the highest risk of subsequent events. These patients had 3 vessel disease ≥ 70% or left main stem stenosis ≥ 50%. This finding was in keeping with other researches which have reported that the presence of obstructive CAD is associated with a poorer prognosis [16–19].

The Duke Prognostic CAD Index was originally derived for important prognostic aspects with a more detailed information of coronary anatomy than the traditional CAD classification of normal, one, two and three-vessel disease, and has been widely used to assess the mortality risk for treatment modalities based on the severity of coronary disease severity [20, 21]. The present study revealed similar prognostic value for predicting all-cause death among suspected CAD patients as those in the CONFIRM registry study, the PROMISE study and a multidetertor CCTA study [12]. However, compared with the Duke Prognostic CAD Index, CAD-RADS classification is more concise and thus is more conducive to enhance communication between interpreting and referring clinicians. Moreover, automated classification of CAD-RADS based on structured reporting systems may improve data quality and then establishing standard databases with education, patient care and research purposes [22–24].

The quantification of CAD scoring systems were initially used as a tool for both clinical practice and scientific investigation by invasive coronary angiography (ICA) [25]. Coronary CTA is a relatively new test that enables noninvasive and direct visualization of the presence and extent of coronary stenosis. Increasing amounts of CAD scoring based on coronary CTA including CACS are used in clinical and research [8, 9, 19, 26]. As a standardized reporting system of CCTA, the primary aim of CAD-RADS is to facilitate the consistent fashion among physicians, including recommendations for further investigations and management. Despite of performing similar as well as traditional CAD classifications and Duke prognostic index in the present, a limitation of CAD-RADS is that only the highest grade of stenosis is considered and more number of groups and complexity than the traditional CAD classifications and CACS. Duke prognostic index based on coronary CTA is also a valuable classification [12]. However, complex classifications also exit than traditional CAD classifications and CACS. Traditional CAD classifications are simple and widely used in clinic. Nevertheless, modifiers with nonevaluable, stent, coronary bypass graft, and high-risk vulnerable plaque features may not be included in the standardized reporting system compared with CAD-RADS.

Despite the import findings and clinical implications for CAD-RADS prognostic value in patients with suspected CAD in the present study, the study had several limitations. First, the study contains a relative larger sample size; however, this was conducted at a single center. In addition, the selection bias may be present with the retrospective nature of this study. Second, the numbers of classes are different according to the classifications that may lead inconsistent of the proportion between complex classifications and simple classifications. Larger samples and multicenter researches are needed to reduce bias. Third, as we restricted our analysis to suspected CAD patients without previously known CAD, CAD-RADS modifiers to describe patients with stents (modifier S), grafts (modifier G), or vulnerable plaque features (modifier V) were not included in the present study. Finally, the present study had limited data on coronary artery calcium (CAC) which was recently shown with high correlation with the presence of obstructive stenosis and suggested to be the main predictor of risk for death by the WDHR (Western Denmark Heart Registry) study [27].

In conclusion, the CAD-RADS classification provided important prognostic information for patients with suspected CAD with noninvasive evaluation, which was non-inferior than Duke Prognostic CAD Index and traditional stenosis-based grading schemes in prognostic value of all-cause mortality. Traditional and simplest CAD classification should be preferable, given the more number of groups and complexity of CAD-RADS and Duke prognostic index, without using more time consuming classification.

## Supplementary Information

**Additional file 1**. Calibration plot of the models.

## Data Availability

The datasets generated during and/or analyzed during the current study are available from the corresponding author on reasonable request.

## Abbreviations

CAD-RADS: Coronary artery disease reporting and data system
CAD: Coronary artery disease
CTA: Computed tomography angiography
ROC: Receiver-operating characteristic
AUC: Area under curve
CVD: Cardiovascular disease
ASCVD: Atherosclerotic cardiovascular disease
CACS: Coronary artery calcification scores
HR: Hazard ratios
CI: Confidence intervals
PCI: Percutaneous coronary intervention
CABG: Coronary artery bypass graft
HRP: High-risk plaque
CAC: Coronary artery calcium
