# Supplementary material for: Prognostic value of CAD-RADS classification by coronary CTA in patients with suspected CAD
Source: BMC Cardiovasc Disord. 2021 Oct 3;21:476. doi: 10.1186/s12872-021-02286-x (PMC8487531; doi:10.1186/s12872-021-02286-x)
Supplement: Supplementary file 1 — Additional file 1. Calibration plot of the models. [file 12872_2021_2286_MOESM1_ESM.docx]

**Fig S1.** Calibration plot of the models. Model 1, Traditional CAD Classification 1; Model 2, Traditional CAD Classification 2; Model 3, Traditional CAD Classification 3; Model 4, Duke Prognostic CAD Index; Model 5, CAD-RADS.


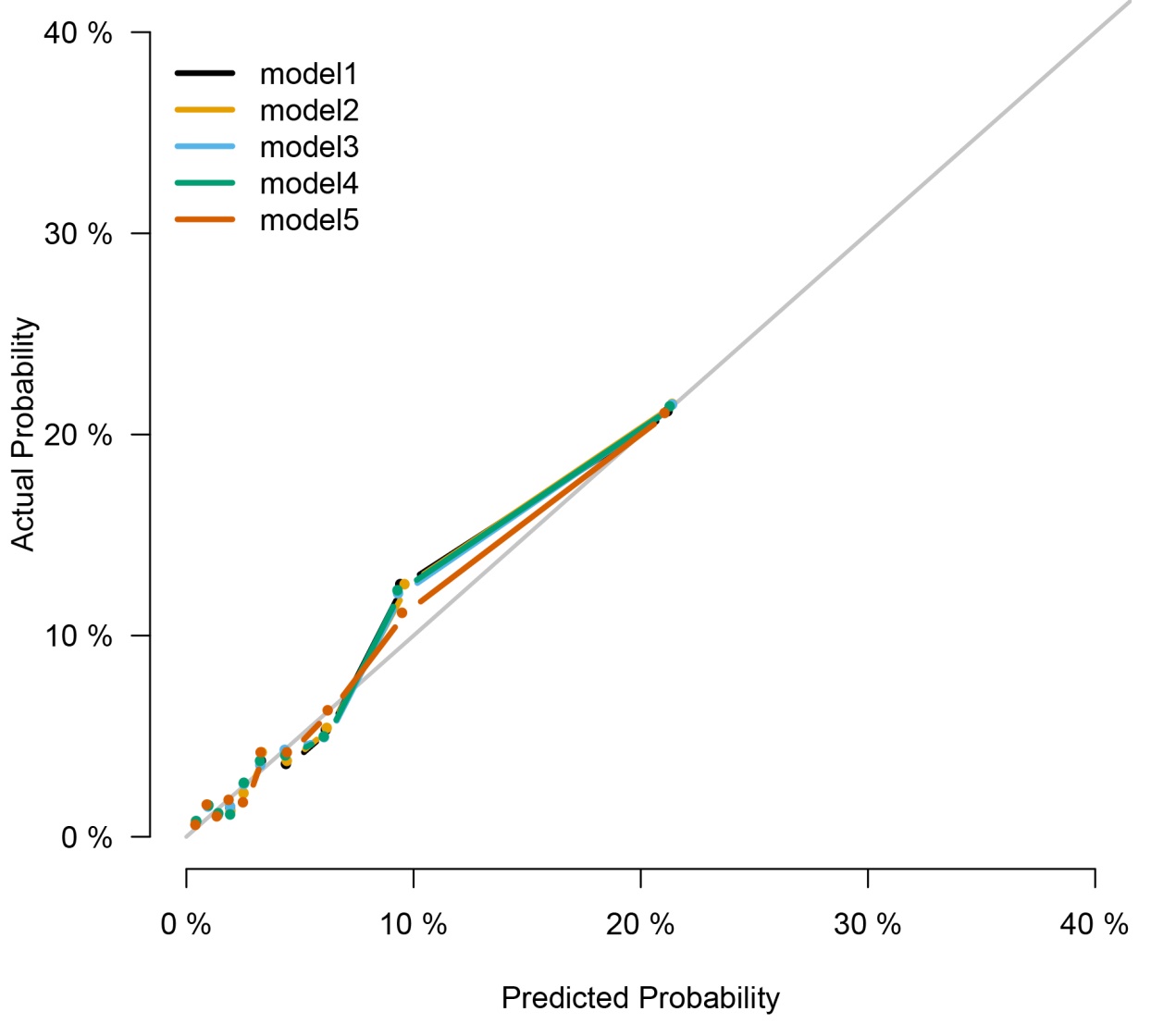


**Supplementary textual file.** Ethical approval of the Institutional Review Board of the Clinical Research Institute at our hospital.


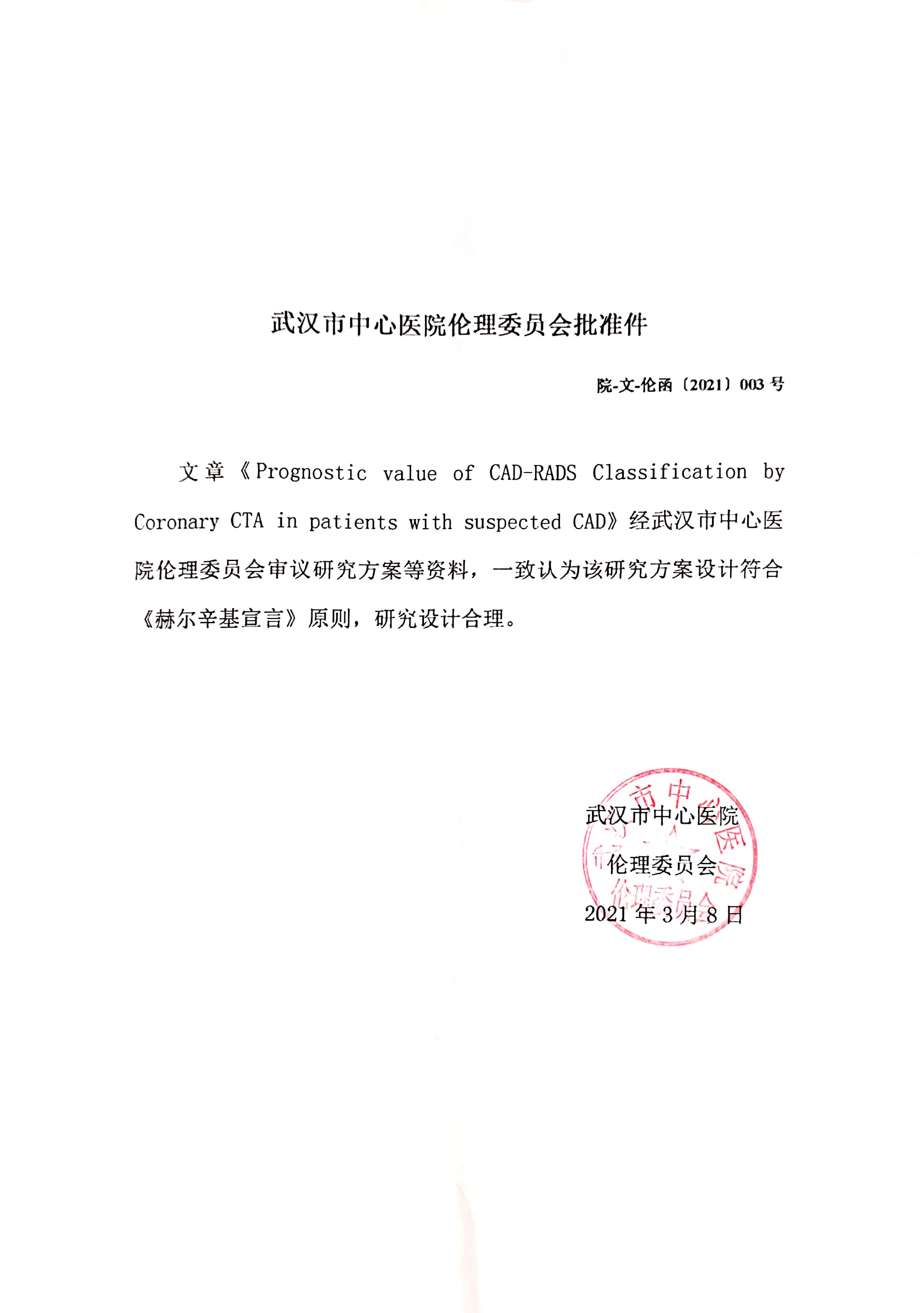


Translation as following:

**Ethics approval by the Institutional Review Board of the Clinical Research Institute at The Central Hospital of Wuhan**

**No.2021-003**

After reviewing the research and other materials, the Institutional Review Board of the Clinical Research Institute at The Central Hospital of Wuhan agreed the design of the research scheme (*Prognostic value of CAD-RADS Classification by Coronary CTA in patients with suspected CAD*) in accordance with the related guidelines and the principles of the Declaration of Helsinki.

Institutional Review Board of the Clinical

Research Institute at The Central Hospital of Wuhan

March 8, 2021
